# Gene Mapping, Cloning and Association Analysis for Salt Tolerance in Rice

**DOI:** 10.3390/ijms222111674

**Published:** 2021-10-28

**Authors:** Xiaoru Fan, Hongzhen Jiang, Lijun Meng, Jingguang Chen

**Affiliations:** 1 School of Chemistry and Life Science, Anshan Normal University, Anshan 114007, China; 2017203043@njau.edu.cn; 2 School of Agriculture, Shenzhen Campus of Sun Yat-sen University, Shenzhen 518107, China; jianghzh23@mail.sysu.edu.cn; 3 Shenzhen Branch, Guangdong Laboratory of Lingnan Modern Agriculture, Genome Analysis Laboratory of the Ministry of Agriculture and Rural Affairs, Agricultural Genomics Institute at Shenzhen, Chinese Academy of Agricultural Sciences, Shenzhen 518120, China; 4 Kunpeng Institute of Modern Agriculture at Foshan, Foshan 528200, China

**Keywords:** salt tolerance, quantitative trait locus (QTL), association analysis, marker-assisted selection (MAS), rice (*Oryza sativa* L.)

## Abstract

Soil salinization caused by the accumulation of sodium can decrease rice yield and quality. Identification of rice salt tolerance genes and their molecular mechanisms could help breeders genetically improve salt tolerance. We studied QTL mapping of populations for rice salt tolerance, period and method of salt tolerance identification, salt tolerance evaluation parameters, identification of salt tolerance QTLs, and fine-mapping and map cloning of salt tolerance QTLs. We discuss our findings as they relate to other genetic studies of salt tolerance association.

## 1. Introduction

Land clearing, excessive irrigation, salt intrusion into coastal zones and sea-level rise has increased soil salinity, and this is now a significant abiotic stress affecting crop production and quality [1]. A total of 6% of the world’s land area and 20% of irrigated agriculture have been affected by soil salinity. Salinity also poses a serious threat to irrigated agriculture [2,3]. The salinity problem in crop production will likely worsen due to the increasing human population [4].

Rice (*Oryza sativa* L.) is a staple food for much of the global population [4,5]. Rice is a salt-sensitive crop and yield can be greatly reduced (by over 50%) when soil salinity exceeds 6 dS/m [6]. Salt tolerance in rice varies as the growth stage does. Rice is salt-sensitive at the seedling stage, moderately salt-tolerant at the vegetative stage, and highly sensitive at the reproductive stage [7]. 

Salt tolerance in rice is controlled by multiple physiological and biochemical reactions, including osmotic stress and ionic stress [3]. Therefore, it is difficult to improve the salt tolerance of rice using traditional breeding methods [7]. Marker-assisted selection (MAS) and genetic engineering technology can accelerate the process of selecting for salt-tolerant rice varieties, but it is difficult to obtain salt-tolerant varieties for crop production by the insertion of single genes [8]. Therefore, it is necessary to simultaneously introduce multiple key genes to improve many pathways in the salt-tolerant regulatory network [8]. It is important to understand the molecular mechanisms and to identify the quantitative trait loci (QTL) and key genes of rice salt tolerance [1,9,10].

Genome-wide QTL analysis has been used to identify salt tolerance-related sites, and this has identified many QTLs related to rice salt tolerance. These studies have provided a foundation for the cloning of salt tolerance genes. The location and cloning of salt-tolerant genes, or QTLs, have promoted molecular-assisted selection breeding in rice. This review summarizes research on rice salt tolerance gene mapping, cloning, and breeding applications to aid breeding of salt-tolerant varieties.

## 2. QTL Analysis of Salt Tolerance in Rice

### 2.1. QTL Mapping Population for Salt Tolerance

Mapping QTLs provides insights in the inheritance mechanisms of the quantitative traits in plants and animals [11]. The mapping populations used for QTL analysis could be divided into permanent populations and temporary populations [11]. In the QTL analysis of salt tolerance in rice, the permanent populations included recombinant inbred lines (RILs) and introgression lines (ILs). RIL population–parent combinations included Kolajoha×Ranjit [12], Jiucaiqing× IR26 [13,14], Changbai10×Dongnong425 [15], Tesanai 2×CB, (Nona Bokra×Pokkali)×(IR4630-22-2-5-1-3×IR10167-129-3-4) [16], IR4630×IR15324 [17], Co39×Moroberekan [18], Milyang23×Gihobyeo [19,20], H359×Acc8558 [21], IR29×Pokkali B [22], Yiai1×Lishuinuo [23], CSR11×MI48 [24], CSR27×MI48 [25], and Dongxiang×NJ16 [7]. IL population–parent combinations included IR64×Tarom Molaii [26], Ilpumbyeo×Moroberekan [27], Minghui86×ZDZ057, Minghui86×Teqing Shuhui527×ZDZ057, Shuhui527×Teqing [28], Lemont×Teqing [29], Pokkali×IR29 [30,31], Teqing×Oryza rufipogon [32], Ce258×IR758 62 [33], Tarome-Molaei×Tiqing [34], Xiushui 09×IR2061 [35], IR64×Binam [36], and Nipponbare×Kasalath [37]. In addition, there are doubled haploid (DH) groups that include IR64×Aucena [38] and Zhaiyeqing 8×Jingxi 17 [39,40]. Some studies also used a set of chromosome segment substitution lines (CSSLs) to detect salt tolerance in seedlings [41]. Mapped salt-tolerant QTLs that have permanent populations could analyze phenotypic variation at multiple points over multiple years. In this way, the identified salt-tolerant QTLs are more stable and not affected by the environment, which was of benefit to map-based cloning and molecular breeding applications. However, most of the permanent populations in the studies were not used for salt tolerance analysis. There was a lack of highly salt-tolerant or salt-sensitive parental varieties. The salt tolerance difference between the parents was small, which was not conducive to the identification of major salt-tolerant sites. Only a few populations were constructed that had salt-tolerant varieties as their parents and used for salt tolerance research, such as Kolajoha×Ranjit [12], Jiucaiqing×IR26 [13,14], (Nona Bokra×Pokkali)×(IR4630-22-2-5-1-3×IR10167-129-3-4) [16], IR29×Pokkali [22], CSR11×MI48 [24], and CSR27×MI48 [25].

Most of the salt-tolerant QTL mapping of rice has used temporary populations. Most of these populations were F_2_ and F_3_ populations, and a few were F_4_, BC_1_F_1_, BC_1_F_2:3_, and BC_2_F_2:3_ populations. The parent populations included Gharib×Sepidroud [42,43], Nona Bokra×Koshihikari [44], Tarommahali×Khazar [45,46], Pokkali×Shaheen Basmati [47], BRRI Dhan40×IR61920-3B-22-2-1 [48], Dongnong425×Changbai10 [15,49], Jiucaiqing×IR36 [50], Sadri×FL478 [51], NERICA-L-19×Hasawi, Sahel 108×Hasawi, and BG90-2×Hasawi [52], IR36×Pokkali [53,54], CSR27×MI48 [55], Cheriviruppu×Pusa Basmati1 [56], and Peta×Pokkali [57]. These populations were used for QTL analysis of salt-tolerant materials such as Gharib, Nona Bokra, Tarommahali, Pokkali, Jiucaiqing, FL478, Hasawi, IR61920-3B-22-2-1, Cheriviruppu, Changbai10, and CSR27 [42,43,44,45,46,47,48,49,50,51,52,55,56].

Some studies used two or more populations simultaneously for salt tolerance QTL analysis. Tiwari et al. [24] identified the salt-tolerant QTLs that had two RIL populations CSR11×MI48 and CSR27×MI48; Cheng et al. and Yang et al. [29,35] used the two-way combination of Xiushui09×IR2061 and Lemont×Teqing; Qian et al. [28] selected Shuhui 527×ZDZ057, Minghui 86×ZDZ057, Shuhui 527×teqing, and Minghui 86×teqing for salt tolerance QTL analysis; Sun et al. [49] used F_3_ and BC_1_F_2:3_ populations of Dongnong 425×Changbai 10 to analyze the dynamic QTL that controls the ion content in rice roots; Bimpong et al. [52] used F_2_ populations of NERICA-L-19×Hasawi, Sahel 108×Hasawi and BG90-2×Hasawi to identify QTLs for salt tolerance in Hasawi. QTL analysis and comparison with multiple mapped populations were conducive to finding salt-tolerant sites that could be stably expressed and less affected by genetic background.

### 2.2. Period and Method of Salt Tolerance Identification

Rice has different tolerances to salt stress at different growth stages [7]. The seedling stage and the reproductive growth stage are salt-sensitive, while the seed germination stage and the vegetative growth stage are more salt-tolerant [7]. Therefore, most of the studies of salt tolerance QTL analysis in rice have been conducted during the seedling and reproductive growth stages [58].

More than half of the QTL studies on rice salt tolerance have used the seedling stage. The methods used for the identification of salt tolerance at the seedling stage were uniform. Rice seedlings were cultivated by hydroponics, and treated with salt at, or near, the three-leaf stage [13,14,16,17,19,20,21,22,23,26,28,29,30,34,35,36,40,43,44,45,46]. For the reproductive growth stage, most rice studies used plants in artificial salt ponds. A small number of studies used rice planted in soil and treated with salt water [24,25,40,51,52,53,54,55,56]. The initial and final salt treatments were different in different studies. Most of the studies transplanted rice to salt ponds in the seedling or tillering stage, where they were grown to maturity. The plants were then scored for agronomic traits and physiological indicators of salt tolerance [24,25,40,51,52,53,54,57]. A few studies analyzed salt tolerance QTL in the seed germination stage, and conducted the germination in a medium with salt as a treatment [13,38,42].

Some studies simultaneously analyzed salt tolerance QTL in two or more growth and development stages. Gu et al. and Pandit et al. [25,57] identified the salt tolerance QTL in the vegetative and reproductive growth stages of rice; Zang et al. [36] identified tolerance in the seedling stage and vegetative growth stage; Ammar et al. [55] analyzed salt tolerance QTLs in seedling, vegetative growth, and reproductive growth stages. These studies helped to identify the genes that control salt tolerance in multiple growth and development stages of rice.

To analyze the influence of plant developmental differences on salt tolerance, some studies used a control group. They analyzed the salt tolerance of the mapping population under the salt and the control treatments at the same time [13,14,24,27,28,36,37,38,40,49,52,53,54,57]. Most of the studies used permanent populations that are homozygous for each strain. A few studies used different tillers from F_2_ populations for different treatments.

### 2.3. Salt Tolerance Evaluation Parameter

The salt tolerance of rice is a complex and comprehensive trait that has various evaluations that differ between development stages. In QTL analysis of rice salt tolerance, the evaluation parameters at the seedling stage can be divided into three categories: morphological, growth and physiological. Morphological parameter analysis evaluates the salt tolerance of the seedlings (score of salt tolerance, SST) by observing the blade tips, leaves, tillers, and the growth inhibition and death of plants after salt stress, and also investigating the survival days of seedling (SDS) after salt stress [14,15,18,19,20,22,23,26,27,28,29,30,33,35,36,43,44,46,47,48,55,59]. Most studies have used the standard evaluation system (SES) proposed by the International Rice Research Institute (IRRI) to evaluate the salt damage level [60]. Some studies modified the evaluation criteria based on experimental materials and experimental design [19,22,26,28,29,30,33,35,36,43,46,47,55,59]. The growth indicators used to evaluate the salt tolerance during the seedling stage include plant height and the fresh and dry weight of shoots and roots [14,17,22,27,43,45,46,47]. There are many physiological parameters for evaluating the salt tolerance of rice, and the indicators for QTL analysis include plant ion content, the concentration of shoot Na^+^ (SNC) and K^+^ (SKC), shoot Na^+^/K^+^ ratio (SNKR), the concentrations of root Na^+^ (RNC) and K^+^ content (RKC), and root Na^+^/K^+^ ratio (RNKR) [14,15,16,17,21,22,26,29,33,34,35,43,44,45,46,47,59]. Some studies also analyzed QTL with the chlorophyll content of seedlings after salt stress [22,43,45].

The evaluation parameters for the salt tolerance of rice seeds during germination include germination rate and germination vigor. Some studies further analyzed growth of the embryo and the radicle of seedlings after germination [13,38,42]. The evaluation parameters during the vegetative growth stage included plant growth and physiological indicators. Most studies analyzed the growth and ion content of the shoot rather than the root [18,25,36,49,50,55,57]. The evaluation during reproductive growth included yield-related agronomic traits, such as the heading date, plant height, tiller number panicles per plant, grains per panicle, seed setting rate, 1000-seed weight, and yield per plant [24,25,40,51,52,55,56,57]. Some studies analyzed the content of Na^+^, K^+^, Ca^2+^, and Cl^−^ in rice leaves or straw after salt treatment in the reproductive growth stage [25,51,53,55]. Some studies included control groups, and they used the absolute value of each evaluation parameter for QTL analysis between the control and comparison groups. They also used the relative value of each salt tolerance trait (treatment/control) or decrease rate ((control–treatment)/control) as an indicator, which was beneficial in reducing the influence of individual plant differences [24,27,49,53,54,57].

### 2.4. Salt Tolerance QTL

We found 52 salt tolerance QTL studies in rice, as shown in Table 1. More than half of the salt-tolerant QTLs were in the seedling stage. Salt-tolerant QTLs at each growth stage were distributed on the 12 rice chromosomes.

The phenotypic contribution rate of a single QTL ranged from 0.02% to 81.56%. A total of 167 QTLs had a contribution greater than 20% and these occupied 22.0% of the total QTLs (Table 1). Salt-tolerant QTLs that have a large contribution to the phenotype were found in the studies that follow. Thomson et al. [22] detected 16 salt-tolerant QTLs which explained more than 20% of the phenotypic variation in the seedling stage. Of these, five QTLs had a contribution exceeding 50%. Qian et al. [28] used four mapping populations and detected 43 QTLs that control SST or SDS in seedlings. The contributions of 12 QTLs were more than 20%. Sabouri et al. [45] identified 32 QTLs that control different growth and physiological indicators of salt tolerance in rice seedlings. Among them, 14 QTLs explained more than 20% of the phenotypic variation. Bimpong et al. [52] detected 75 salt tolerance QTLs in three mapping populations during the reproductive stage, of which about half of the QTLs (37) explained more than 20% of the phenotypic variation. Ammar et al. [55] detected 25 QTLs that had a contribution greater than 10% in the seedling, vegetative growth, or reproductive growth stage, and 22 QTLs among these had a contribution rate over 20%. In these studies, there were 101 salt-tolerant QTLs that had a phenotypic contribution rate exceeding 20%. There were only a few QTLs that had large effects in other studies, and 13 studies had no salt-tolerant QTLs that exceeded a 20% variation. [5,15,17,25,27,30,33,38,47,50,59,61,62].

**Figure 1:**
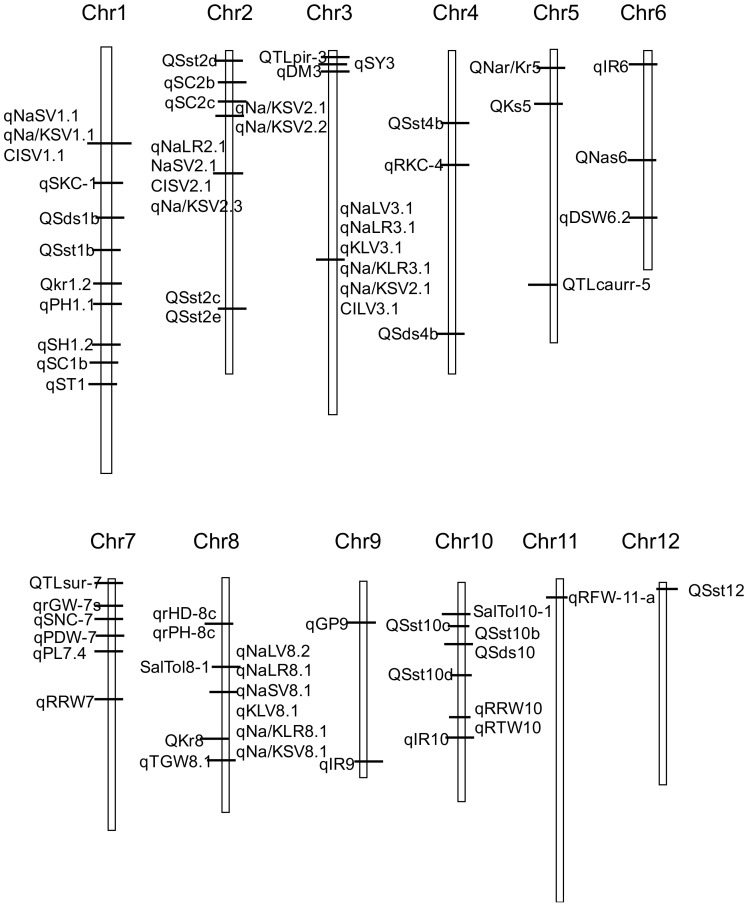
Genetic linkage map showing the location of QTLs for salt tolerance-related traits detected in reports.

We constructed a framework genetic map using 70 QTLs with high PVE in the reports [13,14,19,20,21,34,39,40,42,44,45,48,51] (Figure 1). Figure 1 showed that QTLs related to salt tolerance are distributed on 12 chromosomes, but less on chromosomes 11 and 12.

### 2.5. Fine Mapping and Map-Based Cloning of QTLs for Salt Tolerance in Rice

Because many salt-tolerant rice QTLs have a low phenotypic contribution rate and are difficult to fine-map and clone, relevant research has progressed slowly. However, two QTLs located on the first chromosome, *qSKC-1* and *Saltol*, are suitable for fine-mapping or map-based cloning.

*qSKC-1* is a major QTL that controls the K^+^ content in the shoot. It was detected in the F_2_ population that was constructed by the salt-tolerant variety Nona Bokra and the salt-sensitive variety Koshihikari, and it explained 40.1% of the total phenotypic variation [44]. Ren et al. [67] used the map-based cloning method, followed by fine-mapping of the BC_2_F_2_ population and high-precision linkage analysis of the BC_3_F_2_ population, and they restricted *qSKC-1* within the 7.4 kb chromosome interval and isolated the *qSKC-1* gene. This gene encoded an ion transporter (OsHKT1;5) of the HKT (high-affinity K^+^ transporter) family, which exists in the parenchyma cells of the xylem of rice roots and has the function of specifically transporting Na^+^. This transporter could transport Na^+^ out of the xylem, and transport Na^+^ from the phloem back to the root where it was excreted from the plant through the action of other Na^+^ transporters. This process reduced the Na^+^ content in the shoot, regulated the Na^+^/K^+^ balance in the shoots, and improved rice salt tolerance [68].

Gregorio [69] used AFLP markers to analyze the salt tolerance QTL of the F_8_ recombinant inbred line population of the Pokkali/IR29 combination, and detected a major QTL on rice chromosome 1 that simultaneously controls the Na^+^, K^+^ content and Na^+^/K^+^ ratio in rice. This QTL was named *Saltol*. In the population, the LOD value of the *Saltol* site was greater than 14.5, and the phenotypic contribution rate was 64.3–80.2%. Subsequently, Bonilla et al. [70] used the same population to map *Saltol* to the chromosome between SSR markers RM23 and RM140, and they found that the contribution rates of *Saltol* sites to the Na^+^, K^+^ content and Na^+^/K^+^ ratio were 39.2%, 43.9%, and 43.2%, respectively. Niones and Thomson et al. [22,71] used the near-isogenic lines BC_3_F_4_ and BC_3_F_5_ with IR29 as the background and Pokkali as the donor to confirm the position of the *Saltol* locus. Since the positions of *Saltol* and *qSKC-1* were nearby on the chromosome, and both were responsible for regulating the Na^+^/K^+^ balance of rice under salt stress, Thomson et al. [22] speculated that *Saltol* and *qSKC-1* may encode the same gene (OsHKT1;5).

Some studies conducted fine mapping and cloning on salt-tolerant and salt-sensitive mutants. Lan et al. [72] fine-mapped the seedling salt-tolerant mutant gene *SST* to the 17 kb interval on chromosome 6, and the only predicted gene in this interval is *OsSPL10*, which might be a candidate gene for *SST*. Ogawa et al. [73] and Toda et al. [74] used the salt-sensitive mutants *rss1* and *rss3* to clone the salt-tolerant-related genes *RSS1* and *RSS3*, respectively. *RSS1* participated in the regulation of the cell cycle and was an important factor for maintaining the viability and vigor of meristematic cells under salt stress; *RSS3* regulated the expression of the jasmonic acid-responsive gene, and was involved in maintaining root cell elongation at an appropriate rate under salt stress. Deng et al. [75,76] analyzed the salt-tolerant and salt-sensitive mutants *rst1*, *rss2* and *rss4*. They detected two QTLs (*qSNC-1* and *qSNC-6*) that control the Na^+^ content of aerial parts on chromosomes 1 and 6, which explained 14.5% and 53.3% of the phenotypic variation, respectively. The synergistic alleles were derived from *rss2*.

## 3. Association Analysis of Rice Salt Tolerance

Traditional QTL mapping usually uses markers to perform linkage analysis on the segregating populations derived from the parental cross F_1_. This requires construction of a mapping population with a long cycle, and it has limited mapping accuracy and detectable alleles. Therefore, association analysis based on linkage disequilibrium is more widely used to analyze quantitative traits of plants [77,78]. Association analysis is now commonly applied to the identification of rice salt tolerance genes.

### 3.1. Association Analysis of Salt Tolerance Candidate Genes

To identify salt tolerance QTLs or candidate genes in 180 japonica rice from the European Rice Core Collection (ERCC), Ahmadi et al. [79] used 124 SNP and 52 SSR markers to associate 14 salt tolerance QTLs and 65 salt tolerance candidate genes. They identified 19 gene loci that were significantly associated with one or more salt stress traits. Negrão et al. [80] analyzed 392 rice germplasm resources by EcoTILLING technology, and they found the allele polymorphisms of five salt tolerance candidate genes, which were related to Na^+^/K^+^ balance, signal cascade and stress protection. There were 40 new alleles in the coding sequences of these genes, and 11 SNPs related to salt tolerance in rice were identified by association analysis.

Association analysis identified QTLs, candidate genes, and alleles related to the salt tolerance of rice, and it also revealed different salt tolerance mechanisms of different genotypes of rice. However, none of the rice varieties carried favorable alleles at all salt tolerance loci [79,80].

### 3.2. SSR Association Analysis of Salt Tolerance

SSR markers were used on 300 rice resources to analyze the association between salt tolerance in the seedling stage and tolerance in the whole growth period. Zheng et al. [63] identified the salt tolerance of 342 japonica rice with seedling survival days and shoot Na^+^/K^+^ as evaluation indicators at the seedling stage. They used 160 pairs of SSR markers for salt tolerance association analysis. A total of twelve SSR markers were significantly associated with salt tolerance. A total of nine of the markers were close to the positions of reported salt tolerance QTLs and four markers were in the same position of known salt tolerance-related genes (*OsEREBP1*, *OsABF2*, *HKT1;5* and *OsAHP1*). Cui et al. [81] planted 347 japonica rice on coastal tidal flats, and examined agronomic traits such as heading date, plant height, effective panicle number, grain number per panicle, spikelet fertility, and thousand-grain weight. These traits were used as evaluation parameters for salt tolerance association analysis with 148 SSR markers. The study identified 25 SSR markers linked to rice salt tolerance. These markers are located on 10 chromosomes, except for the fifth and sixth chromosomes, and explained from 4.58% to 31.65% of the phenotypic variation. Among these loci, 10 markers were consistent with, or close to, the positions of reported salt tolerance QTLs on the chromosome.

### 3.3. Salt Tolerance Genome-Wide Association Analysis

Genome-wide association analysis (GWAS) has recently played an important role in discovering genes that regulate plant salt tolerance [82]. Through phenotypic variation analysis, GWAS can analyze the interactions between traits that previously seemed independent, and this can help explain the interactions among potential genes [83,84,85]. 

Kumar et al. [86] identified rice salt tolerance gene loci with 220 rice materials, and they performed an association analysis on 12 agronomic traits related to salt tolerance during the reproductive growth stage and the accumulation of Na^+^ and K^+^ in leaves. They identified 20 SNPs significantly related to leaf Na^+^/K^+^, and 44 SNPs related to other salt tolerance traits. These gene loci explained 5–18% of the phenotypic variation. Zhang et al. [87] used a multiparent advanced generation intercross population, DC1, and identified salt tolerance QTLs by GWAS. There were 7 QTLs delineated from 186 associations that were detected on chromosomes 1, 2, 5 and 9, which explained 7.42–9.38% of the total phenotypic variation. Liu et al. [88] found five known genes (*OsSUT1*, *OsCTR3*, *OsMYB6*, *OsHKT1;4*, and *OsGAMYB*) and two novel genes (*LOC_Os02g49700* and *LOC_Os03g28300*) that were associated with grain yield under salinity stress. Batayeva et al. [89] performed GWAS on 9 seedling salt tolerance traits of 191 japonica rice, and they detected 26 significant loci. Neang et al. [90] used 296 accessions of rice to identify salt-related traits and used 36,901 SNPs to conduct GWAS. They found 13 candidate genes. Yu et al. [91] used 295 accessions and identified 93 candidate genes with high association peaks of salt stress. Cui et al. [92] reported six multi-locus GWAS methods (mrMLM, FASTmrMLM, FASTmrEMMA, pLARmEB, pKWmEB, and ISIS EM-BLASSO), and identified 162,529 SNPs at the seed germination stage for salt tolerance traits with 478 rice accessions. Lekklar et al. [93] conducted a GWAS of salt tolerance using Thai rice accessions, and they found 164 genes co-localized with reported salt quantitative trait loci. These accounted for 73% of the identified loci. Yu et al. [94] performed a GWAS of salt-tolerance-related phenotypes in rice during the germination stage with 295 accessions. They found *OsMADS31*, one of the MADS-box family transcription factors, had down-regulated expression and was predicted to participate in salt stress at the germination stage. Rohila et al. [95] conducted a GWAS of early vigor traits under salt stress with the natural genetic variation in the United States Department of Agriculture rice mini-core collection. They identified 14 salt-tolerant accessions, 6 new loci, and 16 candidate genes that could contribute to salt tolerance breeding. Warraich et al. [96] evaluated 180 rice accessions for salinity tolerance at the reproductive stage by GWAS and 19 associations were identified for Na^+^, K^+^ and Na^+^/K^+^ uptake in leaves and stems. Based on 6,361,920 single nucleotide polymorphisms in 478 rice accessions, Shi et al. [97] identified 22 salt tolerance-associated SNPs based on salt tolerance-related traits. There were seven loci on chromosomes 1, 5, 6, 11, and 12 that were close to six previously identified quantitative gene loci/genes related to salinity tolerance. These studies showed that there were some genes expected to be involved in salt resistance in rice, including a nitrate transporter gene *OsNRT2.1* [97], a MADS-box family transcription factor gene *OsMADS31* [94], a sucrose transport protein gene *OsSUT1*, a transcript factor gene *OsGAMYB*, and some function gene*s OsCTR3*, *OsMYB6* and *OsHKT1;4* [88], etc. With the development of variety resequencing, salt tolerance GWAS has rapidly developed [98,99,100,101,102,103,104,105]. GWAS has exploited the natural variation in root architecture remodeling under salt tolerance to uncover the genetic controls underlying plant responses [83,106]. The studies provide insight into the genetic structure of salt tolerance and are important resources for breeding programs. 

In Table 2, we summarize 25 association analyses of salt tolerance with more than 600 genetic sites related to salt tolerance in rice

## 4. Issues and Prospects

Most studies of rice salt tolerance gene mapping and cloning evaluated the salt tolerance of a specific growth and development stage such as the seedling stage. Few studies have evaluated salt tolerance in multiple growth stages. Due to the differences in the salt tolerance of rice at different growth and development stages, it is necessary to conduct QTL analysis or association mapping for salt tolerance in the different growth stages of rice, especially the seedling stage and reproductive growth stage. This will enable identification of genes that simultaneously control salt tolerance in multiple growth stages. This research could begin by screening salt-tolerant rice germplasm resources during the whole growth period, and then identifying materials that complete their life cycle under salt stress with less impact on yield traits. This approach could be useful for salt-tolerant gene mapping, cloning, and breeding.

Hundreds of QTLs related to salt tolerance have been identified but the progress of follow-up work on fine-mapping and map-based cloning of genes has been slow. One reason is the lack of salt-tolerant rice varieties in the parental combinations. A small difference in salt tolerance between the two parents may only result in a small contribution to the phenotype of the identified QTLs and disturb the fine-mapping by the genetic background. However, most of salt-tolerant QTL mapping studies involved a single time period or a single-year phenotypic identification, and there was a lack of QTL stability. Some salt-tolerant QTLs with a high phenotypic contribution rate (above 20%) could be difficult to fine-map and clone due to poor genetic stability. Therefore, it is necessary to select strong salt-tolerant rice varieties for salt-tolerant QTL mapping, and also to test the stability of salt-tolerant QTLs multiple times or by multi-year multi-point experiments for gene cloning and breeding.

For mining salt-tolerant genes for salt-tolerant germplasm resources, traditional QTL analysis methods could be combined with mutant construction, screening, and association analysis. Association analysis, especially GWAS, is now commonly used for the analysis of complex traits of plants, and it aids understanding of the genetic basis and differentiation of salt tolerance in rice. Using QTL mapping and GWAS, the genetic basis of many complex quantitative traits has been analyzed and many QTL segments or loci have been located. With the development of genome sequencing technology and bioinformatics, using the reference genome sequence information of the corresponding species could help determine the candidate genes related to the target trait and narrow the range of candidate genes [110,111]. Moreover, GWAS is useful for marker-assisted selection (MAS) of rice varieties suitable for cultivation in salinized fields. 

With the development of molecular marker technology, MAS technology is now widely used in crop breeding, thus providing a new way to accelerate the genetic improvement of rice salt tolerance. MAS could select target traits in early generations, accelerate the breeding process, and aggregate multiple beneficial genes at the same time to improve breeding efficiency [112]. A QTL for salt tolerance in rice that is frequently used in MAS breeding is *Saltol*, which is located on chromosome 1 [113]. *Saltol* QTL is a major QTL associated with the Na^+^/K^+^ ratio and salinity tolerance at the seedling stage in rice. Several genes have been reported in the *Saltol* QTL (*LEA*, *CaMBP*, *V-ATPase*, *GST*, *OSAP1* zing finger protein and transcription factor *HBP1b*) that were salinity sensitive and regulated between the genotypes [114,115,116]. Most studies were performed using marker-assisted backcrossing (MABC) technology in India, the Philippines, Bangladesh, Thailand, Vietnam and Senegal [117,118,119]. Some *Saltol* introgression lines cultivated by MAS, such as BR11-SalTol and BRRI dhan28-SalTol, have been tested in salt damaged coastal areas in the Philippines, Bangladesh, India, and Vietnam [113,120]. Bimpong et al. [113] found that compared with traditional breeding, MAS breeding can shorten the germplasm improvement time by four to seven years.

## Figures and Tables

**Table 1:** Identified QTL for salt tolerance in rice.

Stage	Parents for Cross	Population Type	Evaluation Parameter for Salt Tolerance	PVE%	QTL	High-PVE QTL	Reference
Germination stage	IR64×Azucena	DH	GR, seedling root length, seedling dry mass, seedling vigor	13.5–19.5	7	0	[38]
	Jiucaiqing×IR26	RIL	GR, RL, SH	6.5–43.7	7	4	[13]
	Gharib×Sepidroud	F_2_/F_2:4_	GR, germination percentage, radicle length, plumule length, coleoptile length, radicle fresh weight, plumule fresh weigh, radicle dry weight, plumule dry weight, coleoptile fresh weight, coleoptile dry weight	10.0–21.9	17	2	[42]
	9311×japonica	CSSL	Survival rate	5.1–93.2	4	-	[41]
Seedling stage	Dongnong425×Changbai10	BC_2_F_2_/BC_2_F_2:3_	SST, SNC, SKC, RNC, RKC	6.45–17.95	13	0	[63]
	O. rufipogon×O. Sative	ILs	SDS, STT	2–8	10	-	[64]
	(Nona Bokra×Pokkali)×(IR4630-22-2-5-1-3×IR10167-129-3-4)	RIL	SNC, SKC, SNKR	-	4	-	[16]
	IR4630×IR15324	RIL	SNC, SKC, SNKR, total Na^+^ and K^+^, SDW	6.4–19.6	11	0	[17]
	Milyang 23×Gihobyeo	RIL	SST	9.2–27.8	2	1	[19]
	Milyang 23×Gihobyeo	RIL	SST	9.1–27.8	2	1	[20]
	H359×Acc 8558	RIL	SNC	1.68–45.39	13	3	[21]
	IR29×Pokkali	RIL	SNC, SKC, RKC, RNKC, SH, chlorophyll content, seedling survival rate, initial and final SST	6–67	27	16	[22]
	Yiai1×Lishuinuo	RIL	Dead rate of leaf and seedling	8.65–27.20	6	1	[23]
	IR64×Tarom Molaii	IL	SST, SDS, SKC, SNC, RKC, RNC	-	23	-	[26]
	Ilpumbyeo×Moroberekan	IL	The reduction rate of fresh and dry weight, leaf area and SH	10.2–13.9	8	0	[27]
	Shuhui527×ZDZ057, Minghui86×Teqing, Minghui86×ZDZ057, Shuhui527×Teqing	IL	SST, SDS	8.17–42.18	43	12	[28]
	Lemont×Teqing	IL	SST, SDS, SKC, SNC	-	36	-	[29]
	Pokkali×IR29	IL	SST	4.00-18.42	6	0	[30]
	Ce258×IR75862, ZGX1×IR75862	IL	SST, SDS, SKC, SNC	5.13–13.75/3.73–8.26 *	18/2 *	0	[33]
	Tarome-Molaei×Tiqing	IL	SNC, SKC, SNKR, RNC, RKC, RNKR	9.0–30.0	14	5	[34]
	Xiushui 09×IR2061-520-6-9	IL	SST, SDS, SKC, SNC, SKNR	5.14–18.89/2.60–14.30 *	26/21 *	0	[35]
	Zaiyeqing8×Jingxi17	DH	SDS	10.2–38.4	10	2	[39]
	Nona Bokra×Koshihikari	F_2_/F_3_	SDS, SNC, SKC, RNC, RKC, Na^+^ and K^+^ in root, SDW	12.4–48.5	11	3	[44]
	Tarommahalli×Khazar	F_2_/F_3_	Survival rate, chlorophyll content, SH, RL, leaf area, the weight of stem and root, total Na^+^ and K^+^ in shoot, SNKR	9.03–38.22	32	14	[45]
	Tarommahali×Khazar	F_2_/F_3_	STR, DM, Na^+^ content, K^+^ content, Na^+^/K^+^	9.03–20.90	14	1	[46]
	Pokkali×Shaheen Basmati	F_2_/F_3_	SST, SH, SDW, SFW, SNC, SKC, SNKR, RNC, RKC, RNKR	4.89–10.55	22	0	[47]
	BRRI Dhan40×IR61920-3B-22-2-l	F_2_	SST	12.5–29.0	3	2	[48]
	Jiucaiqing×IR26	RIL	RNKR, SH, SDW, RDW	7.8–23.9/- *	15/5 *	2	[14]
	Jiucaiqing×IR26	RIL	RKC, SNC, SKC, SST	8.5–18.9/- *	13/9 *	0	[59]
	Tesanai 2×CB	RIL	SDS	1.5–11.6	4	0	[61]
	Tesanai 2×CB	RIL	SDS, SDW, RDW, SNC, SKC, SKNR	4.4–15.0	31	0	[62]
	Teqing×*Oryza rufipogon*	IL	SST, relative SDW, RDW and total plant dry weight	8–26	15	3	[32]
Vegetative growth stage	Co39×Moroberekan	RIL	Content of Na^+^ in shoot, SNKR, fresh weight of stem, moisture content of leaf	11.0–26.3	14	3	[18]
	Nipponbare×Kasalath	IL	SH, SDW, number of tillers	12–41	31	11	[37]
	Dongnong425×Changbai10	BC_1_F_2_/BC_1_F_2:3_, F_2_/F_3_	RNC, RKC, RNKR, relative RNC, relative RKC, relative RNKR	3.61–27.9	50	4	[49]
	CSR10×Taraori Basmati	F_3_	Relative growth rate, SNKR, visual salt-injury symptoms	25.6–31.3	14	-	[65]
	Jiucaiqing×IR26	F_2_	SST, SNKR, SDW	6.7–19.3	7	0	[50]
Reproductive growth stage	CSRll×MI48, CSR27×MI48	RIL	Sensitivity index of grain yield stress	-	55	-	[24]
	Zhaiyeqing 8×Jingxi 17	DH	Effective tiller number, thousand-grain weight, PH, heading date, number of grains per panicle	7.9–40.1	24	3	[40]
	Sadri×FL478	F_2_	Heading date, PH, length and number of panicles, dry weight of straw, number of fertile and sterile spikelets per plant, total number of spikelets per plant, yield per plant, spikelet fertility, thousand grain weight	4.2–30.0	37	1	[51]
	NERICA-L-19×Hasawi, Sahel108×Hasawi, BG90-2×Hasawi	F_2_	SST, PH, TN, heading date, panicle number per plant, panicle sterility rate, grain number per ear, thousand-grain weight, yield per plant	6.5–49.5	75	37	[52]
	IR36×Pokkali	F_2_	Content of Na^+^ and Ca^2+^, absorption rate of Ca^2+^, relative content of Na^+^, K^+^ and Ca^2+^, relative ion content, relative absorption rate of Na^+^, K^+^, Ca^2+^ and Na^+^/K^+^	7.69–26.33	14	3	[53]
	IR36×Pokkali	F_2_	PH, TN, number of effective tillers, panicle weight, panicle length, number of spikelets panicle, number of unfilled grains panicle, number of grains panicle, panicle fertility, days of 50% flowering, days to maturity, grain length, grain width, grain length–width ratio, grain yield, thousand-grain weight, straw yield, harvest index	11.52–81.56	6	1	[54]
	Cheriviruppu×Pusa Basmati 1	F_2_	PH, TN, panicle length, yield, biomass, pollen fertility, Na^+^ content in flag leaf, Na^+^/K^+^	3.8–48.7	24	5	[56]
	HHZ×Budda, HHZ×Gang46B	BC_2_F_5_	Grain weight, spikelet number, thousand-grain weight, seed fertility	4.7–90.6	22	1	[66]
	Sahel108×Hasawi, NERICA-L-19×Hasawi, BG90-2×Hasawi	F_2_	Days to flowering/heading, PH, TN, panicle sterility, grain yield, yield per plant, yield-component data for each plot, salt tolerance score	7.3–31.9	75	-	[52]
	Tarommahalli×Khazar	F_2_/F_3_	PH, TN, number of full grains, number of empty grains, length and number of panicle, biomass	8.76–26.83	12	3	[56]
Multiple growth stages	CSR27×MI48	RIL	Vegetative growth period: content of Na+ in stem, content of K^+^ and Cl^−^ content in leaf	5.86–8.55	4	0	[25]
			Reproductive growth period: content of Na^+^, K^+^ in straw, Na^+^/K^+^ in straw, spikelet fertility stress sensitivity index	7.22–14.05	5	0	
	IR64×Binam	IL	Seedling stage: SST, SDS, SKC, SNC	-	13	-	[36]
			Vegetative growth period: PH, panicle number, fresh weight	-	22	-	
	CSR27×MI48	F_2_/F_3_	Seedling stage: SST	14.38	1	0	[55]
		F_2_	Vegetative growth period: Na^+^, Cl^−^ content in leaf and stem, K^+^ content in stem, Na^+^/K^+^ in leaf and stem	11.13–55.72	17	15	
		F_2_	Reproductive growth period: content of Na^+^, K^+^ and Cl^−^ in leaf, Na^+^/K^+^ in leaf	26.26–52.63	7	7	
	Peta×Pokkali	BC_1_F_1_	Vegetative growth stage: SST, SFW, SDW, Na^+^ content	-	4	-	[57]
			Reproductive growth stage: weight of stem and leaf, PH, TL, effective panicle, number, panicle weight, main panicle length, grain weight, seed setting rate	-	11	-	

Note: DH: double haploid. RIL: recombinant inbred lines. IL: introgression line. CSSL: chromosome segment substitution line. F_2_: second filial generation. F_2_/F_3_: second and third filial generation. F_2_/F_2:4_: F_2_ generation and F_2_ derived fourth filial generation. BC_1_F_1_: first backcross generation. BC_2_F_5_: twice backcross and four selling generation. BC_1_F_2_/BC_1_F_2:3_: twice backcross and one selling generation, and one backcross and one selling derived third filial generation. BC_2_F_2_/BC_2_F_2:3_: twice backcross and one selling generation, and twice backcross and one selling generation derived fourth filial generation. High-PVE QTL: the number of QTL with contribution > 20%. GR: germination rate. SDS: survival days of seedling. SST: score of salt tolerance. SNC and SKC: the concentrations of Na^+^ and K^+^ in shoots. SNKR: shoot Na^+^/K^+^ ratio. RNC and RKC: the concentrations of Na^+^ and K^+^ in roots. RNKR: root Na^+^/K^+^ ratio. SH: shoot height, RL: root length, SDW: shoot dry weight. SFW: shoot fresh weight. RDW: root dry weight. PH: plant height. TN: tiller number, STR: standard tolerance ranking, DM: dry matter weight. * indicate major QTL/epistatic QTL.

**Table 2:** Association analysis of salt tolerance in rice.

Stage	Population Size	Maker Type	QTL	Traits	Reference
Generation stage	478	SNP (6.36M)	11	GR, germination index, vigor index, germination time, and imbibition rate	[97]
	295	SNP (1.65M)	12	GR, germination energy, germination index, SH, RL	[94]
	184	SNP (788K)	8	RL under control condition, alkaline stress and relative RL	[101]
Seedling stage	32	SSR (64)	28	Salt tolerance level	[107]
	342	SSR (160)	12	SST, SNC, SKC, RNC and RKC	[15]
	533	SNP (700K)	20	Relative growth rate, transpiration use efficiency and transpiration rates	[108]
	295	SNP (1.65M)	25	Leaf width, SH, RL, total dry weight	[91]
	235	SNP (30K)	27	Tiller number, SH, RL, SDW, RDW, RDW/SDW, leaf area, SNKR	[79]
	306	SNP (200K)	58	SNC, SKC	[103]
	203	SNP (68K)	26	Shoot Na^+^ and K^+^ content, standard evaluation score, percentage of damage, SDW	[89]
	708	SNP (3.45M)	41	SDS, SSI	[88]
	162	SNP (3.2M)	9	SSI, SDW, RDW, SDS	[95]
	176	SSR (154)	13	Salinization damage grade, SNC, SKC, SNKR	[109]
	221	SNP (55K)	7	SES, SDS, SH and RL under salt treatment, relative SH and RL, SDW and RDW after salt treatment, relative SDW and RDW, relative biomass.	[87]
	181	SNP (32K)	54	SH, SFW and SDW under control, salt stress conditions, relative SH, SFW and SDW	[98]
	295	SNP (788K)	8	SST, SNC, SKC, SNKR	[100]
	664	SNP (3M)	21	SH, RL, SFW, SDW, RFW, RDW, salt tolerance level	[105]
Vegetative growth stage	104	SNP (112K)	200	Photosynthetic parameters and cell membrane stability	[93]
	296	SNP (44K)	11	Na^+^ and Cl^−^ of leaf blades, Na^+^ and Cl^−^ sheath:blade ratios, SES	[90]
	179	SNP (21K)	26	SES, chlorophyll content, water content, Na^+^ and K^+^ contents, SNKR	[99]
	96	SNP (50K)	23	SH, RL, SFW, SDW, RFW, RDW, RNC, SNC, RKC, SKC, RNK, SNKR, RNKR	[104]
Reproductive growth stage	220	SNP (6K)	64	SNKR, PH, TN, spikelet fertility, unfilled or filled grains, yield	[86]
	347	SSR (148)	25	Salinity tolerance index	[81]
Multiple growth stages	180	SSR (150)	28	Na^+^, K^+^, Ca^2+^, Mg^2+^ content in stem and leaves, grain yield and SSI	[96]
	208	SNP (395K)	20	Generation stage: GR.Seedling stage: SH, RL	[102]

Note: GR: germination rate. SDS: survival days of seedling. SSI: salt stress injury score. SST: score of salt tolerance. SNC and SKC: the concentrations of Na^+^ and K^+^ in shoots. SNKR: shoot Na^+^/K^+^ ratio. RNC and RKC: the concentrations of Na^+^ and K^+^ in roots. RNKR: root Na^+^/K^+^ ratio. SES: standard evaluation system score. SH: shoot height, RL: root length, SDW: shoot dry weight. SFW: shoot fresh weight. RDW: root dry weight. RFW: root fresh weight. PH: plant height. TN: tiller number.

## Data Availability

All of the data generated or analyzed during this study are included in this published article.
